# Buffalo Academy of Medicine

**Published:** 1901-03

**Authors:** Julius Ullman


### Society Proceedings.

### BUFFALO ACADEMY OF MEDICINE.

*Section on Medicine, January 9, 1901.*

REPORTED BY JULIUS ULLMAN, M. D., Secretary.

THE fourth regular meeting of the Section of Medicine was held in the Library Building, Thursday evening, January 9, 1901, Dr. ELI H. LONG presiding.

Dr. D. A. MORRISON read a paper, entitled,

REMINISCENT OBSERVATIONS OF MALARIA.

Dr. Morrison introduced the subject by referring to the past played by the plasmodium malaria and its classification into forms producing the quotidian, quartan and estivo-autumnal types of the disease. He remarked that up to the present time no record of its successful cultivation has been published, but that of late much new light has been developed on its transmissibility through the mosquito —the genus anopheles acting as the intermediary host. The writer spoke of his residence in the City of Buffalo for the past sixteen years, during which time he never had occasion to treat a single case of malaria, and compared this condition to that of the first years of his practice in the State of Iowa, where 50 per cent. of all cases treated by him were due to malaria and its sequences.

The geographical distribution of malaria in temperate climes and its constant prevalence in the tropics, because the soil, moisture and seasons with other conditions are almost perpetual for favoring its generation.

Dr. Morrison described the conditions of soil, climate and geological strata in that portion of Iowa in which he saw his patients, and showed how conducive they were for the propagation of the disease. During June and July of 1881 the preponderance of cases seen were the quartan forms, and with these cases many, especially children, developed a cerebro-spinal meningitis. In the following year the tertian variety of fever was prevalent. During the autumn of the first year there were many cases of remittent malaria, with a few cases of typhoid, while in the autumn of the second year an epidemic of typhoid prevailed, with a few cases of remittent fever.

In this territory there were many squatter settlers, those coming from Sweden and Norway seemed more susceptible to the infection. With the appearance of the tertian variety of malaria great swarms of gnats, mosquitoes and other insects appeared on the uplands during sunset. They would collect each variety by itself, usually on the west and northwest slopes of small knolls of land in close proximity to swamps. In the early morning, the whole surface of the plain would be enveloped with a fog or low lying mist and as the sun advanced this would clear away.

Malaria was so common that the inhabitants accepted their miserable sufferings with the stolidity of fatalists. Many of the more intelligent inhabitants regarded the malady as due to a poison or miasm arising from the marshy lands, and that the poison was inhaled by them. Upon this theory they cultivated walls of sunflowers, hoping thus to filter this poison, and thus be afforded an immunity. It seemed that this really did act as a barrier, for fewer cases was seen among those thus protected. The sunflower evidently offered an obstruction to insect life.

The clinical symptoms of malaria in this locality were always typical; never atypical. The quotidian chills always occurred between 12 m. and 4 p.m. If pernicious in character, between 3 and 6 p.m. It was not uncommon to see victims while on errands of business to the village in hot summer months, bring blankets and overcoats and when the hour approached for the expected paroxysm, they would cover themselves after mounting some convenient box on the sunny side of the street and await the chill.

In all protracted cases there was enlargement of the spleen, liver, anemia, emaciation and loss of general vitality. It was noted that young cattle seemed to be infected with this disease. Young cattle were often found standing in the hot sun between 11 a.m. and 1.00 p.m. close to a wire fence, with backs humped and hair standing erect, suffering to all appearances a rigor or chill. This occurred every forty-eight hours, and although grazing was of the best, they would emaciate, and many died later of what was termed "black leg," which was characterised by edema of the extremities. The animals slaughtered in the fall showed enlargement of the liver and spleen.

The Indian who took up his abode in this locality was not exempt from malaria and cases of the intermittent type were seen in their camps. In the pernicious forms of malaria, the comatose occurred most frequently; the algid form was rare and the hemorrhagic form never presented itself.

In the treatment quinine was the specific. It was used during the attacks and as a prophylatic. The Swedes and Norwegians had regular family jugs, containing one gallon of equal parts of alcohol and water with a solution of quinine, representing five grains to the ounce. The Indian medicine man used roots, herbs, barks, and hearts of wood trees. A sample of a decoction made from the red heart of the ironwood tree was tested and alkaloid was found, which resembled quinine in taste, color, and other qualities.

DISCUSSION.

Dr. JULIUS ULLMAN said that it is becoming generally accepted as a fact that malaria is rarely if ever seen in people who live in Buffalo. In other words, those rare cases of malaria which appear are imported. Quinine, therefore, in large doses, is now fortunately prescribed with greater precision in this city. He recalled his experience of acting assistant surgeon at the Leiter General Hospital at Chickamauga in 1898. Each day the errors of the volunteer surgeons in camp and division hospitals were revealed. Fearful doses of quinine were given to typhoid fever patients, with the mistaken idea that malaria was prevalent. Some of the patients when brought to the hospital were absolutely deaf. Many came in a dying condition. It was a pity to think that many patients who died might have been saved, had the diagnosis of the case been made earlier and had they not lost valuable time by overdosing with quinine with the mistaken idea that malaria existed where it did not.

Dr. Ullman regarded the observation made by Dr. Morrison of having seen a disease in cattle, resembling in the paroxysm and the post mortem findings malaria in man as of importance, and hoped that further proof would in time accumulate on this statement. He referred to Texas fever or bovine malaria in cattle, in which there is a parasite somewhat similar to that in the human blood found in the red blood corpuscles, and here the intermediary host has been demonstrated as the tick; so also the potiosma of birds, in which the mosquito also acts as the intermediary host.

Dr. Singer stated that he has not seen a case of malaria since he has practised in Buffalo.

Dr. Dowd reported a case of hemaglobinuria, showing a sample of urine therefrom. Briefly the history was as follows:

J. C., aged 25., M. About Christmas, 1899, noticed for the first time that the urine seemed to be very bloody; this has continued ever since, with the exceptions of a day or two at a time when it will be clear. Just previous to the appearance each morning, the hands and feet become very cold, also the back which becomes numb-like. At the same time he becomes very jaundiced, has fever and the pain extends up the back into the head, which aches violently, necessitating him to leave his work. The liver is very large. As the symptoms abate, headache, and the like, gradually disappear. Never had venereal disease, and there is no history of tuberculosis. Examination of urine shows: specific gravity, 10.30, very acid, opacity marked, of a blood-red character; indican increased; large number of granular casts, apparently casts of amorphous urates. No blood, no pus, few leucocytes; sediment consists of red colored débris, evidently broken down cellular elements, epithelium and amorphous urates.

Two years ago while a soldier in the West Indies (St. Lucia), had a severe attack of malaria. Has taken large amount of quinine, but trouble still continued. With the use of quinine, arsenic and iron the condition (blood colored urine) improved at once; in three weeks having but two attacks of discolored urine, accompanied by chills, and the like. No examination for the malarial plasmodium was made, the writer taking the opinion of a Montreal physician, who found these parasites present about a year ago. The spectroscope was not used, Dowd depending upon the microscope for the diagnosis.

Bastianelli says: this practically never occurs except in infections with the estivo autumnal parasite.

DISCUSSION.

Dr. GROVER WENDE thinks that the interesting case described by Dr. Dowd is one of paroxysmal hemoglobinuria, especially because the doctor remarked that the patient had taken large amounts of quinine without result, which is sufficient evidence to exclude malaria.

Dr Wende offers the suggestion that ice be applied to the skin because cold in this case is a causative factor.

Dr. MORRISON stated that malarial hemoglobinuria is associated with the attacks and paroxysms early in the disease. In view of the prolonged history of malaria (two years) without this complication makes some other cause than malaria suggestive.

---

*Section on Ophthalmology, Otology, Rhinology and Laryngology, January 21, 1901.*

REPORTED BY FREDERICK H. MILLENER, M. D., Secretary pro tem.

The fifth regular meeting of this section was held on January 21, 1901. The meeting was called to order at 9.05 p.m., by Dr. FRANK WHITHILL HINKEL, chairman, eighty-seven Fellows being present. The secretary, Dr. GEORGE F. COTT being ill, the chairman appointed Dr. Millener, secretary, pro tempore. The minutes of the previous meeting were approved.

Dr. HINKEL then introduced the speaker of the evening, Dr. GEORGE T. STEVENS, of New York, who addressed the section on

THE POSE OF THE BODY AS RELATED TO THE TYPE OF THE CRANIUM AND THE DIRECTION OF THE VISUAL PLANE.

See page 576.

The paper was discussed by Dr. LUCIEN HOWE, who stated that, if he understood the trend of the argument made by Dr. Stevens, it was this: (1) That by means of the tropometer we are enabled to ascertain in certain cases that the muscles of the eyes tend to direct the visual axis above the horizontal plane or below it, as the case may be; (2) this being ascertained, the author has found by measurement that these conditions belong to certain forms of the skull and, (3) accompanying this cranial formation, there is a certain expression of the face and pose of the head, which are characteristic. Or the proposition may be reversed, and we may say that certain facial expressions are so characteristic as to enable the skilled observer to diagnosticate the position of the eyes with respect to the horizontal plane, and therefore the condition of the muscles. Dr. Howe asked if he had correctly interpreted the author's views.

Dr. Stevens replied that it was substantially the statement of his contention.

Dr. Howe, continuing, remarked that he was sorry to be obliged to take issue with the author, and stated that we all appreciate his lifelong interest in the subject of the ocular muscles, and use generally of the nomenclature concerning them which he has suggested, but, though he comes so far to present this paper, he will not be surprised at the criticism of it, for he is a member of another society of which Dr. Howe is also a member, in which every statement is thoroughly criticised, and if a vulnerable point is presented in any one of the papers offered, the other members immediately take up the cudgels, and, if possible, throw the author on the defensive. With this spirit of scientific doubt and inquiry, therefore, he wished to take up briefly the three points in the doctor's argument.

The first is concerning the clinical value of the tropometer. He stated that he not only had had one of these instruments himself for three or four years, but also an opportunity to use another whenever desired at the Buffalo Eye and Ear Infirmary. After a measurement of a large number of cases, he was convinced that it is not possible to draw from it any such exact data.

The fact is that the muscular effort made by the patient is not always the same. For example, when he looked through the instrument and asked the patient to look up, he found the amount of elevation to be thirty degrees, and if the patient be urged suddenly the elevation passed to thirty-five or again perhaps to forty or more. Moreover, this difference varied on different days. In a word, after a long trial, it had been found unreliable.

Thinking, however, that the fault might be his own, he had brought here this evening one of Stevens's tropometers, and would be glad to have the test made before the members present, and have these statements as to inaccuracy refuted by actual trial, if that were possible.

As to the second point in the author's argument, he wished simply to remark that it has long been known that the form of the cranium bore a certain relation to the form of the eyes and, therefore, that part of the paper was not new.

Donders and Javal called attention to this in the later sixties and it became a part of ophthalmological literature. De Wecker corroborated the observations in a communication made by him in 1869, to the Anthropological Society of Paris, and in 1881, Landolt reviewed the subject admirably in an article in the *British Medical Journal.* In 1892, at the meeting of the American Medical Association, Dr. Stevens presented the paper which, with modifications, he has repeated this evening.

In this paper tonight, he has called attention to this well known relation between the form of the skull and the shape of the eye, but has gone a step further and elaborated the third point.

This third point, which he makes, is that in these peculiarities in the formation of the skull, the muscles of the eyes direct the visual axes above or below the horizontal plane, as indicated in a characteristic manner, in which the head of the individual is thrown backward or forward. Or, reversing this part of the argument, given, such a position of the head, it is possible from that to recognise a certain condition of the muscles?

Now, in regard to this third proposition, it is true that in a rough way some slight indication may be given as to the condition of the muscles, especially in cases of real paralysis. But, if the statement made by Dr. Stevens will bear the test of practice, then it can be easily verified.

Dr. Howe also said that in this audience of about seventy-five or eighty, he happened to know of at least three individuals, who have a high degree of heterophoria of the kind mentioned. And if the facial expression and pose of the head of such person or persons is so diagnostic, then he would ask the author to pick out these or any other similar cases from among the rest. There need be no question as to the accuracy of the choice, for Dr. Howe purposely brought here a tropometer, a clinoscope, a phorometer, and all the apparatus necessary, and they were in the adjoining room. It, therefore, was only fair to ask for a demonstration of the fact, and if proven, Dr. Howe would be prompt to acknowledge that his views, and not Dr. Stevens's were wrong.

Dr. STEVENS in reply said: In reference to the statement that Donders and other European writers have treated of the subject which has been presented this evening, I may state in the most emphatic manner that Donders never wrote a line which indicates that he had ever thought upon this subject. Nor did any European writer ever discuss this subject in any of its phases before it was presented by myself. The fact that someone may have written of microcephalic and distorted skulls in association with undeveloped eyes, has no possible relation to the subject here discussed.

As to the statement that one has attempted to use the tropometer and has failed to acquire useful information, the same may be said of the microscope or of any other technical instrument. Skill is required in the use of the tropometer, as it is in the use of the microscope. No one gains useful information from the use of scientific instruments who does not bring to their use scientific methods and an unprejudiced mind.

Dr. Alvin A. Hubbell moved a vote of thanks to our distinguished colleague, stating that those who know him best appreciate his efforts. Many of us know very little about this subject, and he may startle us, but Dr. Stevens has startled the world before. Seconded by Dr. Howe and carried unanimously.

Meeting adjourned at 11.15 p.m.

---

*Section on Obstetrics and Gynecology, January 22, 1901.*

Reported by WM. G. RING, M. D., Secretary.

The regular monthly meeting of this section was held at the Library Building on Tuesday evening, January 22d, at 8.30 p.m., and was called to order by the chairman, Dr. A. J. Colton.

The minutes of the previous meeting were read and approved.

Dr. Stephen Y. Howell read a paper entitled,

THE KIDNEYS AND THEIR RELATION TO OPERATIONS.

DISCUSSION.

Dr. M. D. Mann.—This subject has interested me for years and I think what Dr. Howell says is true. If there is danger in the use of the ordinary anesthetics it behooves us to use only the best anesthetic. In five cases of my own, all fatal, ether was used. One patient had had Bright's disease previously. Operation was followed by suppression of urine and death. We tried different anesthetics. The urine was examined for albumin and casts. After ether 50 per cent. had albumin and casts persisting from four to ten days.

Dr. Roswell Park uses chloroform; I use ether– same results. I have been careful to carry out the line of treatment, Dr. Howell suggested—namely, hot air baths, water and alkalies before operation. Notwithstanding all this care you cannot take all precautions. I think there is just as much danger from chloroform as from ether—from chloroform patients die on the table; from ether, later. When gas is used it takes less ether. I do not believe that if the kidneys are healthy we will get bad results.

In examination of the urine I like to estimate the total solids for the twenty-four hours. Ethridge gives a table of the total solids in the urine for a given weight and age. I do not believe urea is the toxic agent in urine. If the kidneys are diseased both chloroform and ether will work harm. I had a patient with cancer of the cervix with hemorrhages. I removed the uterus and she passed urine the following morning. After second catheterisation she never passed a drop. Applied treatment, but patient died. Post mortem showed me kidney atrophied almost to nothing; the other was a large white kidney containing in its pelvis seven or eight stones. This question of anesthetics is an extremely important one and brings us down to the question of cocanisation of the cord.

Dr. E. A. Smith.—I wish to say that the paper is a timely one; it carries a warning to us all. Have been assured in so-called emergency operations by attending physician that everything was all right, but have taken urine for examination and found differently. He cited a case were both sugar and albumin were found. Another case diagnosticated as colecystitis, proved to be a kidney full of pus. Removed kidney by anterior route, took a sample of the urine and found it loaded with pus. I agree with Dr. Mann as to precautions to be taken.

In one case where urea was found to be deficient, the operation was postponed and the patient sent home for treatment, preliminary to operation, which was performed later with good results. With some cases, when kidney is in bad condition, have seen good results when ether was used. I would also recommend the use of salt solution, per rectum, after operation.

Dr. F. W. McGuire asked if urea was toxic; if so, what importance attached to its absence?

Dr. Ludwig Schröter asked if absence of albumin and casts was evidence that kidneys were in good condition? I think not, and cited a case.

S. Y. Howell, in closing the discussion, said he was pleased with the discussion of his paper, although it was not complete enough to cover all questions. Creatin and creatinin are toxic, but the greatest toxicity is in the inorganic solids. But few urinometers are accurate and in taking the specific gravity, many fail to take into consideration the temperature of the urine tested. Often times it is necessary to make repeated examinations of the urine.

The whole question is an interesting one. The question of ingestion of nitrogenous food, age, tissue metabolism, and the like, should all be taken into consideration, as none of the tests are absolute.

Meeting adjourned at 10 p.m. Members present, 20.

---

*Section on Surgery, February 5, 1900.*

Reported by J. HENRY DOWD, M. D., Secretary pro tem.

The Surgical Section of the Academy of Medicine was called to order at 8.45 p.m. by the chairman, Dr. Marshall Clinton. Dr. J. Henry Dowd was appointed secretary pro tem.

The minutes of the last meeting were not read on account of the absence of the secretary.

The secretary, pro tem., read a telegram of regret from Dr. Samuel Alexander, of New York, who was scheduled to present a paper before the section this evening. Owing to the serious illness of his brother, Dr. Alexander said it was impossible for him to be present.

Dr. Marcel Hartwig showed a case on which he had operated for continuous vomiting, the man vomiting everything he put in his stomach for the past three months. A diagnosis of stricture of the pyloris was made and such proved to be correct at operation. The Doctor could not say whether cancerous or not. Since operation there has been no vomiting and the patient gained 7½ pounds in one week. Soon after the patient recovered from the anesthetic, it was found he had a paralysis of the right arm. Dr. Hartwig did not attempt to explain the cause, excepting to say that a person unconscious from an anesthetic is insensible to pain, but he should be treated gently just the same, and in his opinion the paralysis in question was due to some one grabbing the patient forcibly with fingers in the axilla, and in this way has damaged some of the nerves in that region.

Dr. Dowd showed a foreign body removed from the bladder by the perineal route. The patient was referred by Dr. W. G. Gerber and complained of frequency of urination, the act being accompanied by great pain and a free flow of blood after the urine. As a cause for the condition the young man—aged 25 and every appearance of suffering from some degeneracy of the nervous system—said he experienced some pain in urinating about December 4, 1900, and thinking there was something in the urethra proceeded to operate upon himself with a wax taper—commonly used for lighting gas, at least eighteen inches long—which slipped from his grasp and had entered the bladder. The patient was sent to the hospital, perineal section made, and with but very little trouble the substance was withdrawn. There is no doubt that the true reason for the patient using the wax taper in the urethra was to titilate the caput gallinaginis, and thus produce an orgasm, as when coming from the anesthetic he tried to masturbate.

Dr. Chauncey P. Smith read a paper entitled,

CONSERVATIVE SURGERY OF THE EXTREMITY.

He reported several results, which owing to the damaged condition of the parts should be considered exceptionally good. Emphasis was put on the statement, never to put a drainage-tube in a joint, as by the pressure exerted on the cartilages it was liable to cause their death. He cited several cases of compound fracture near the ankle joint, in which after thoroughly scrubbing of the wound with salt solution and approximation of the parts, the wound was closed tightly and good results obtained in every case. Continuous immersion in hot salt solution was recommended where the part was on the point of gangrene; he stating that even where the tissues were beginning to turn, good results had been obtained in the prevention of the gangrenous process.

The case of Mr. Hood, the man who was bitten by a bear at the zoo, was shown. Although there was a destruction of most of the parts near the wrist-joint, there was sufficient blood supply left to insure life to the hand and the member, although quite badly crippled, was much better than none at all.

Dr. Kenerson read a paper entitled,

SURGICAL COMPLICATIONS OF TYPHOID FEVER.[1]

He spoke from his experience while a contract surgeon in the U. S. Army, at Fort Meyer, Va., dwelling at length upon cases of peritonitis, intestinal hemorrhage, pleurisy, parotitis, epididymitis, orchitis and phlebitis, that had come under his care.

The third paper of the evening was presented by Dr. Le Breton, entiled,

1. To be published.

OPERATIVE TREATMENT OF TUBERCULAR LYMPHOMATA OF THE NECK.

The subject was dealt with in detail, laying special stress on the incisions and the care of the bloodvessels in the surrounding parts. for this work the author advised the incisions of Hartley and Dowd (C. N. of N.Y.,) both of which he described most minutely.

Dr. C. P. Smith made a few remarks, in which he drew a plan, showing how easily the gland could be removed from Scarpa's triangle.

Meeting adjourned at 10.30. Attendance, 17.

---

Suggestions on the Manner of Using Protargol.—Having passed the experimental stage it may now be safely asserted, that protargol is one of the most important additions to the materia medica of recent years. Aside from its general use in the treatment of gonorrheal affections, it has to a great extent displaced nitrate of silver in diseases of the eye, ear, nose and throat. To obtain uniformily good results, attention has been lately drawn to the importance of exercising proper care in making the solutions, a point which has been specially emphasised by Professor Neisser. A clear and satisfactory solution can be secured in any one of the following ways: Stir the protargol powder into a thick and smooth paste with a little cold water, and then add the bulk of the fluid. This should be done in a glass or china vessel, using a glass rod; if in a mortar, the latter as well as the pestle should be slightly moistened with a few drops of glycerine. Protargol may also be readily dissolved by dusting the powder evenly upon the surface of the water and allowing the fluid to stand without stirring for about ten minutes. It is very essential that only *cold* water should be used in making the solutions, as with warm water the drug is to some extent decomposed, and then becomes less active and may cause irritation; for the same reason the solutions should be preserved in dark colored yellow bottles. In acute gonorrhea the average strength of the solutions ranges from 1 to 10 grains to the ounce; in chronic urethritis, up to 30 grains; in diseases of the eyes, ears, nose and throat, 10 to 60 grains; as an application to wounds and ulcers, 1 to 2 per cent. solutions and 5 per cent. ointments are in use. Unlike nitrate of silver, portargol does not stain the skin even in concentrated solution. The solutions commonly employed in gonorrhea also do not produce stains of the clothing, or if they do, only cause slight discoloration, which can be easily removed with warm soap water; stains by stronger solutions, if recent, can be removed with soda and ammonia; if old, by the action of peroxide of hydrogen in the presence of ammonia.—*Therapeutic Suggestions.*

